# Dimethyl [(4-fluoro­phen­yl)(6-methoxy­benzothia­zol-2-ylamino)meth­yl]phospho­nate

**DOI:** 10.1107/S1600536809015384

**Published:** 2009-05-07

**Authors:** Yan-Ping Hong, Bao-An Song, Xin-Chen Shangguan

**Affiliations:** a College of Food Science and Engineering, Jiangxi Agricultural University, Nanchang 330045, People’s Republic of China; b Key Laboratory of Green Pesticide and Agricultural Bioengineering, Ministry of Education, Guizhou University, Guiyang 550025, People’s Republic of China

## Abstract

In the mol­ecule of title compound, C_17_H_18_FN_2_O_4_PS, both the benzene ring with its conjunction C atom and the benzothia­zole ring with its conjunction N atom are close to planar (the maximum deviations are 0.0267 and 0.0427 Å for the benzene and benzothiazole rings, respectively), the dihedral angle between the planes of the benzothia­zole and benzene rings is 119.05 (3)°. The mol­ecular packing is stabilized by inter­molecular N—H⋯O, C—H⋯N and C—H⋯F hydrogen bonding, and by C—H⋯π and π–π stacking inter­actions [centroid–centroid distances = 2.99 (2), 2.96 (3), 2.88 (2) and 3.773 (4) Å].

## Related literature

For the biological activity of α-amino­phospho­nate derivatives, see: Kafarski & Lejczak (2001 ▶); De Lombaert *et al.* (1995 ▶); Du *et al.*, (1999 ▶). For activities of α-amino­phospho­nate derivatives containing an F atom and benzothia­zole or iaoxazole units, see: Yang *et al.* (2005 ▶); Song *et al.* (2005 ▶); Jin *et al.* (2006 ▶). For related structures, see: Fang *et al.* (2009 ▶); Yang *et al.* (2005 ▶); Jin *et al.* (2006 ▶); Song *et al.* (2005 ▶); Alvarez *et al.* (2005 ▶); Chen & Li (1987 ▶); Li *et al.* (2008 ▶).
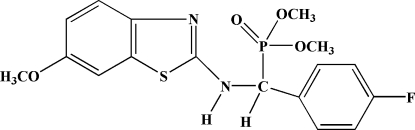


## Experimental

### 

#### Crystal data

C_17_H_18_FN_2_O_4_PS
*M* *_r_* = 396.36
Monoclinic, 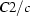

*a* = 19.761 (5) Å
*b* = 15.781 (4) Å
*c* = 14.395 (4) Å
β = 122.109 (3)°
*V* = 3802.4 (16) Å^3^
*Z* = 8
Mo *K*α radiation
μ = 0.29 mm^−1^
*T* = 296 K
0.30 × 0.26 × 0.22 mm

#### Data collection

Bruker APEXII area-detector diffractometer
Absorption correction: multi-scan (*SADABS*; Sheldrick, 1996 ▶) *T*_min_ = 0.919, *T*_max_ = 0.939
9669 measured reflections
3428 independent reflections
2477 reflections with *I* > 2σ(*I*)
*R*_int_ = 0.039

#### Refinement

*R*[*F* ^2^ > 2σ(*F* ^2^)] = 0.041
*wR*(*F* ^2^) = 0.115
*S* = 1.04
3428 reflections
238 parameters
H-atom parameters constrained
Δρ_max_ = 0.22 e Å^−3^
Δρ_min_ = −0.26 e Å^−3^

### 

Data collection: *APEX2* (Bruker, 2004 ▶); cell refinement: *SAINT* (Bruker, 2004 ▶); data reduction: *SAINT*; program(s) used to solve structure: *SHELXS97* (Sheldrick, 2008 ▶); program(s) used to refine structure: *SHELXL97* (Sheldrick, 2008 ▶); molecular graphics: *SHELXTL* (Sheldrick, 2008 ▶); software used to prepare material for publication: *SHELXTL*.

## Supplementary Material

Crystal structure: contains datablocks I, global. DOI: 10.1107/S1600536809015384/at2769sup1.cif

Structure factors: contains datablocks I. DOI: 10.1107/S1600536809015384/at2769Isup2.hkl

Additional supplementary materials:  crystallographic information; 3D view; checkCIF report

## Figures and Tables

**Table 1:** Hydrogen-bond geometry (Å, °)

*D*—H⋯*A*	*D*—H	H⋯*A*	*D*⋯*A*	*D*—H⋯*A*
N1—H1⋯O2^i^	0.86	1.99	2.793 (3)	156
C16—H16*C*⋯F1^ii^	0.96	2.51	3.2496	133
C15—H15*B*⋯*Cg*1^iii^	0.96	2.99	3.608 (4)	123
C16—H16*B*⋯*Cg*3^iv^	0.96	2.96	3.549 (4)	121
C17—H17*C*⋯*Cg*1^v^	0.96	2.88	3.625 (3)	136

Symmetry codes: (i) 
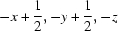
; (ii) 
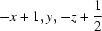
; (iii) 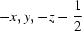; (iv) 
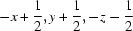
; (v) 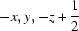. *Cg*1 and *Cg*3 are the centroids of the S1/N2/C4–C6 and C9–C14 rings, respectively.

**Fig. 1.**
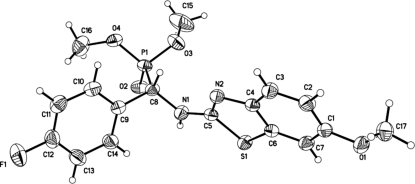
The structure of the title compound, showing as 50% probability displacement ellipsoids.

**Fig. 2.**
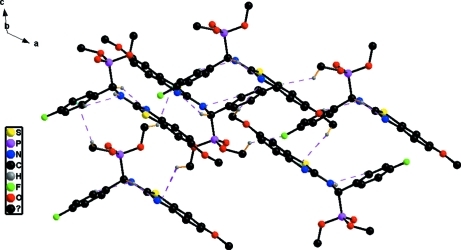
A packing view of the title compound. N—H···O, C—H···O, C—H···N and C—H···F hydrogen bonds, C—H···π and π-π stacking interactions are shown as dashed lines.

## supplementary crystallographic information

### Comment

α-Aminophosphonate derivatives, as isosteres of natural aminocarboxylic acids, have attracted much attention in medicinal and pesticide chemistry in recent years due to their wide range of biological activities (Kafarski *et al.*, 2001; De Lombaert *et al.*, 1995; Du *et al.*, 1999). In our previous work, a plenty of α-aminophosphonate derivatives containing fluorine atom and benzothiazole or iaoxazole moieties had been synthesized and some of them showed fungicidal (Yang *et al.*,2005) and antitumor activities (Song *et al.*, 2005; Jin *et al.*, 2006). And some crystals of α-aminophosphonate derivatives containing dialkyl had been reported in our previous works. However, It is noteworthy that a cystal of α-aminophosphonate containing dimethyl moiety was obtained for the first time. We report herein the crystal structure of the title compound.

As illustrated in Fig. 1, both the benzene ring with its conjunction carbon atom C8 and the benzothiazole ring with its conjunction nitrogen atom N1 are fairly planar, the dihedral angle between the planes of the benzothiazole group (C1–C7/N2/S1) and the benzene ring (C9–C14) is 119.05 °. The P atom exhibits a distorted tetrahedral configuration because the bond angles of O2—P1—O3 = 116.07 (11) ° and O2—P1—O4 = 114.04 (10) ° are significantly larger than that of O3—P1—O4 = 103.65 (10) °. The P1—C8 = 1.807 (2) Å is similar to the corresponding P—C value of 1.809 (3) Å found in diisopropyl [(benzoylamino)(phenyl)methyl] phosphonate (Fang *et al.*, 2009) and a little shorter than normal P—C single bond length of 1.850 Å (Chen *et al.*, 1987). The C5—N1 = 1.355 (3) Å is remarkably shorter than normal C—N 1.471 (3) Å (Alvarez *et al.*, 2005) and close to the C = N = 1.343 (2) Å, similar to the corresponding bond length of 1.358 (3) Å of the 2-(1-piperidinyl)-1,3-benzothiazole (Alvarez *et al.*, 2005). Meanwhile, the S1—C6 and S1—C5 with bond lengths of 1.742 (2) Å and 1.760 (2) Å, respectively, are shorter than the typical C—S of 1.811 (9) Å (Li *et al.*, 2008). These indicate that the N1 atom and benzothiazole ring form a considerable delocalization of the electron density in which the N1 atom is *sp*^2^ hybridized. The molecular packing is consolidated through weak inter/intra molecular N—H···O, C—H···N, and C—H···F hydrogen bonding, C—H···π and π-π stacking interactions (Table 2, Fig. 2, *Cg*1 = ring (S1/N2/C4—C6); *Cg*3 = ring(C9–C14)). C—H···π interactions of methyl H atoms are established towards the π-systems of neighboring aromatic groups from 4-fluorophenyl and five rings of 6-methoxybenzothiazol-2-ylamino, with centroid-to-centroid distance are 2.99 (2), 2.96 (3) and 2.88 (2) Å, respectively. In the π-π stacking interactions between two close-by phenyl rings of 4-fluorophenyl, tThe centroid-to-centroid distance is 3.773 (4) Å.

### Experimental

A mixture of 2-amino-6-methoxylbenzothiazole (0.9010 g, 5 mmol) and 4-fluorobenzaldehyde (0.6205 g, 5 mmol) and 20 ml toluene in a 50 ml dry flask affiliated a water separator was refluxed and stirred for 6 h. Toluene was removed by water separator and then dimethylphosphate (0.88 g, 8 mmol) was added into the flask, the reaction mixture was refluxed in a nonsolvent condition for 5 h. Residue was washed with water, filtered, dried, and crystallized three times from ethanol yielding title compound as colorless solid. The crystal suitable for X-ray analysis was obtained by slow evaporation of an anhydrous ethanol at room temperature over a period of ten days.

### Refinement

H atoms were placed in calculated positions and were treated as riding on the parent C atoms with C—H = 0.93 - 0.987 Å; N—H = 0.86 Å, and with *U*_iso_(H) = 1.2*U*_eq_(C) for CH_2_, CH, NH, and 1.5*U*_eq_(C) for CH_3_ .

### Crystal data

**Table:**

C_17_H_18_FN_2_O_4_PS	*F*(000) = 1648
*M**_r_* = 396.36	*D*_x_ = 1.385 Mg m^−^^3^
Monoclinic, *C*2/*c*	Mo *K*α radiation, λ = 0.71073 Å
Hall symbol: -C 2yc	Cell parameters from 5837 reflections
*a* = 19.761 (5) Å	θ = 2.8–27.9°
*b* = 15.781 (4) Å	µ = 0.29 mm^−^^1^
*c* = 14.395 (4) Å	*T* = 296 K
β = 122.109 (3)°	Block, colourless
*V* = 3802.4 (16) Å^3^	0.30 × 0.26 × 0.22 mm
*Z* = 8	

### Data collection

**Table:**

Bruker APEXII area-detector diffractometer	3428 independent reflections
Radiation source: fine-focus sealed tube	2477 reflections with *I* > 2σ(*I*)
graphite	*R*_int_ = 0.039
φ and ω scans	θ_max_ = 25.2°, θ_min_ = 1.9°
Absorption correction: multi-scan (*SADABS*; Sheldrick, 1996)	*h* = −23→23
*T*_min_ = 0.919, *T*_max_ = 0.939	*k* = −18→18
9669 measured reflections	*l* = −15→17

### Refinement

**Table:**

Refinement on *F*^2^	Primary atom site location: structure-invariant direct methods
Least-squares matrix: full	Secondary atom site location: difference Fourier map
*R*[*F*^2^ > 2σ(*F*^2^)] = 0.041	Hydrogen site location: inferred from neighbouring sites
*wR*(*F*^2^) = 0.115	H-atom parameters constrained
*S* = 1.04	*w* = 1/[σ^2^(*F*_o_^2^) + (0.0545*P*)^2^ + 1.1262*P*] where *P* = (*F*_o_^2^ + 2*F*_c_^2^)/3
3428 reflections	(Δ/σ)_max_ < 0.001
238 parameters	Δρ_max_ = 0.22 e Å^−^^3^
0 restraints	Δρ_min_ = −0.26 e Å^−^^3^

### Special details

**Table:**

Geometry. All e.s.d.'s (except the e.s.d. in the dihedral angle between two l.s. planes) are estimated using the full covariance matrix. The cell e.s.d.'s are taken into account individually in the estimation of e.s.d.'s in distances, angles and torsion angles; correlations between e.s.d.'s in cell parameters are only used when they are defined by crystal symmetry. An approximate (isotropic) treatment of cell e.s.d.'s is used for estimating e.s.d.'s involving l.s. planes.
Refinement. Refinement of *F*^2^ against ALL reflections. The weighted *R*-factor *wR* and goodness of fit *S* are based on *F*^2^, conventional *R*-factors *R* are based on *F*, with *F* set to zero for negative *F*^2^. The threshold expression of *F*^2^ > σ(*F*^2^) is used only for calculating *R*-factors(gt) *etc*. and is not relevant to the choice of reflections for refinement. *R*-factors based on *F*^2^ are statistically about twice as large as those based on *F*, and *R*- factors based on ALL data will be even larger.

### Fractional atomic coordinates and isotropic or equivalent isotropic displacement parameters (Å^2^)

**Table:**

	*x*	*y*	*z*	*U*_iso_*/*U*_eq_	
S1	0.07360 (4)	0.28313 (4)	0.06124 (5)	0.0510 (2)	
P1	0.20273 (4)	0.09893 (4)	−0.09266 (5)	0.0483 (2)	
N2	0.06939 (11)	0.11776 (12)	0.06276 (16)	0.0477 (5)	
C5	0.10708 (13)	0.18124 (14)	0.05470 (17)	0.0421 (5)	
C4	0.00865 (13)	0.14714 (14)	0.07713 (18)	0.0445 (5)	
C6	0.00177 (13)	0.23554 (14)	0.07921 (17)	0.0438 (5)	
C7	−0.05321 (14)	0.27298 (16)	0.09682 (19)	0.0524 (6)	
H7	−0.0568	0.3317	0.0981	0.063*	
C1	−0.10315 (14)	0.22182 (16)	0.11256 (19)	0.0506 (6)	
C3	−0.04236 (14)	0.09767 (16)	0.0913 (2)	0.0571 (7)	
H3	−0.0395	0.0390	0.0890	0.069*	
C2	−0.09822 (15)	0.13484 (16)	0.1089 (2)	0.0563 (6)	
H2	−0.1326	0.1009	0.1184	0.068*	
N1	0.16956 (11)	0.17525 (12)	0.04083 (17)	0.0524 (5)	
H1	0.1923	0.2209	0.0382	0.063*	
C8	0.19929 (13)	0.09419 (14)	0.03023 (19)	0.0452 (6)	
H8	0.1605	0.0505	0.0196	0.054*	
C9	0.27995 (14)	0.06879 (14)	0.12740 (18)	0.0452 (6)	
C11	0.37489 (18)	−0.04111 (18)	0.2316 (2)	0.0659 (8)	
H11	0.3884	−0.0982	0.2439	0.079*	
C10	0.30051 (17)	−0.01584 (16)	0.1484 (2)	0.0560 (6)	
H10	0.2630	−0.0567	0.1049	0.067*	
C14	0.33614 (15)	0.12855 (17)	0.1936 (2)	0.0625 (7)	
H14	0.3237	0.1859	0.1810	0.075*	
C12	0.42812 (17)	0.0196 (2)	0.2955 (2)	0.0684 (8)	
C13	0.41102 (17)	0.1034 (2)	0.2788 (3)	0.0751 (9)	
H13	0.4489	0.1435	0.3238	0.090*	
F1	0.50137 (11)	−0.00510 (13)	0.37934 (15)	0.1064 (7)	
O1	−0.15552 (10)	0.26361 (12)	0.13111 (15)	0.0658 (5)	
C17	−0.20925 (16)	0.21423 (19)	0.1466 (2)	0.0707 (8)	
H17A	−0.2437	0.1822	0.0813	0.106*	
H17B	−0.2409	0.2511	0.1617	0.106*	
H17C	−0.1794	0.1762	0.2071	0.106*	
O2	0.24876 (11)	0.17048 (11)	−0.09456 (13)	0.0612 (5)	
O4	0.23331 (10)	0.01050 (10)	−0.10441 (13)	0.0555 (4)	
O3	0.11234 (11)	0.09795 (13)	−0.18384 (15)	0.0752 (6)	
C16	0.31296 (19)	−0.0033 (2)	−0.0807 (3)	0.0886 (10)	
H16A	0.3292	0.0444	−0.1056	0.133*	
H16B	0.3138	−0.0536	−0.1177	0.133*	
H16C	0.3490	−0.0101	−0.0031	0.133*	
C15	0.0861 (2)	0.1088 (3)	−0.2974 (3)	0.1293 (16)	
H15A	0.1166	0.1532	−0.3040	0.194*	
H15B	0.0305	0.1236	−0.3386	0.194*	
H15C	0.0938	0.0569	−0.3254	0.194*	

### Atomic displacement parameters (Å^2^)

**Table:**

	*U*^11^	*U*^22^	*U*^33^	*U*^12^	*U*^13^	*U*^23^
S1	0.0591 (4)	0.0372 (4)	0.0646 (4)	−0.0040 (3)	0.0382 (3)	0.0005 (3)
P1	0.0512 (4)	0.0440 (4)	0.0452 (3)	−0.0112 (3)	0.0226 (3)	−0.0035 (3)
N2	0.0461 (11)	0.0395 (11)	0.0582 (12)	−0.0059 (9)	0.0282 (10)	−0.0038 (9)
C5	0.0426 (13)	0.0393 (13)	0.0389 (12)	−0.0061 (10)	0.0180 (11)	−0.0032 (9)
C4	0.0451 (13)	0.0396 (14)	0.0455 (13)	−0.0073 (10)	0.0219 (11)	−0.0049 (10)
C6	0.0455 (13)	0.0416 (14)	0.0425 (12)	−0.0052 (11)	0.0221 (11)	−0.0035 (10)
C7	0.0572 (15)	0.0428 (14)	0.0582 (15)	−0.0044 (12)	0.0313 (13)	−0.0054 (11)
C1	0.0490 (14)	0.0532 (16)	0.0514 (14)	−0.0053 (12)	0.0278 (12)	−0.0090 (11)
C3	0.0579 (16)	0.0412 (15)	0.0777 (18)	−0.0081 (12)	0.0398 (15)	−0.0053 (12)
C2	0.0549 (15)	0.0535 (17)	0.0678 (16)	−0.0135 (12)	0.0374 (14)	−0.0064 (12)
N1	0.0529 (12)	0.0390 (11)	0.0741 (14)	−0.0097 (9)	0.0396 (11)	−0.0039 (10)
C8	0.0488 (14)	0.0367 (13)	0.0547 (14)	−0.0089 (11)	0.0306 (12)	−0.0056 (10)
C9	0.0538 (14)	0.0431 (14)	0.0464 (13)	−0.0045 (11)	0.0319 (12)	−0.0008 (10)
C11	0.087 (2)	0.0563 (18)	0.0570 (16)	0.0167 (16)	0.0403 (17)	0.0113 (14)
C10	0.0756 (18)	0.0459 (16)	0.0494 (14)	−0.0029 (13)	0.0352 (14)	−0.0002 (11)
C14	0.0611 (17)	0.0487 (16)	0.0647 (17)	−0.0037 (13)	0.0247 (15)	−0.0009 (13)
C12	0.0643 (18)	0.079 (2)	0.0573 (16)	0.0179 (16)	0.0290 (15)	0.0111 (15)
C13	0.0598 (18)	0.070 (2)	0.0721 (19)	−0.0062 (15)	0.0192 (16)	−0.0051 (15)
F1	0.0756 (12)	0.1209 (17)	0.0865 (13)	0.0313 (11)	0.0186 (10)	0.0189 (11)
O1	0.0625 (11)	0.0652 (12)	0.0842 (13)	−0.0062 (9)	0.0488 (11)	−0.0148 (10)
C17	0.0617 (18)	0.087 (2)	0.0781 (19)	−0.0073 (15)	0.0467 (16)	−0.0076 (15)
O2	0.0787 (12)	0.0477 (10)	0.0626 (11)	−0.0209 (9)	0.0412 (10)	−0.0049 (8)
O4	0.0680 (11)	0.0442 (10)	0.0622 (10)	−0.0108 (8)	0.0399 (9)	−0.0105 (8)
O3	0.0579 (11)	0.0853 (14)	0.0560 (11)	−0.0091 (10)	0.0125 (10)	0.0023 (10)
C16	0.093 (2)	0.086 (2)	0.125 (3)	0.0037 (19)	0.084 (2)	0.003 (2)
C15	0.120 (3)	0.149 (4)	0.054 (2)	−0.030 (3)	0.003 (2)	0.009 (2)

### Geometric parameters (Å, °)

**Table:**

S1—C6	1.742 (2)	C9—C14	1.381 (3)
S1—C5	1.760 (2)	C9—C10	1.382 (3)
P1—O2	1.4593 (17)	C11—C12	1.357 (4)
P1—O3	1.5560 (19)	C11—C10	1.372 (4)
P1—O4	1.5653 (18)	C11—H11	0.9300
P1—C8	1.807 (2)	C10—H10	0.9300
N2—C5	1.290 (3)	C14—C13	1.386 (4)
N2—C4	1.401 (3)	C14—H14	0.9300
C5—N1	1.355 (3)	C12—C13	1.355 (4)
C4—C3	1.373 (3)	C12—F1	1.359 (3)
C4—C6	1.404 (3)	C13—H13	0.9300
C6—C7	1.374 (3)	O1—C17	1.427 (3)
C7—C1	1.384 (3)	C17—H17A	0.9600
C7—H7	0.9300	C17—H17B	0.9600
C1—O1	1.368 (3)	C17—H17C	0.9600
C1—C2	1.379 (3)	O4—C16	1.439 (3)
C3—C2	1.388 (3)	O3—C15	1.438 (4)
C3—H3	0.9300	C16—H16A	0.9600
C2—H2	0.9300	C16—H16B	0.9600
N1—C8	1.449 (3)	C16—H16C	0.9600
N1—H1	0.8600	C15—H15A	0.9600
C8—C9	1.513 (3)	C15—H15B	0.9600
C8—H8	0.9800	C15—H15C	0.9600
			
C6—S1—C5	88.47 (10)	C14—C9—C8	121.5 (2)
O2—P1—O3	116.07 (11)	C10—C9—C8	120.1 (2)
O2—P1—O4	114.04 (10)	C12—C11—C10	118.1 (3)
O3—P1—O4	103.65 (10)	C12—C11—H11	120.9
O2—P1—C8	113.48 (10)	C10—C11—H11	120.9
O3—P1—C8	101.64 (11)	C11—C10—C9	121.7 (3)
O4—P1—C8	106.64 (10)	C11—C10—H10	119.1
C5—N2—C4	109.72 (19)	C9—C10—H10	119.1
N2—C5—N1	125.0 (2)	C9—C14—C13	120.3 (3)
N2—C5—S1	116.95 (17)	C9—C14—H14	119.9
N1—C5—S1	118.01 (17)	C13—C14—H14	119.9
C3—C4—N2	126.0 (2)	C13—C12—C11	122.6 (3)
C3—C4—C6	118.4 (2)	C13—C12—F1	119.0 (3)
N2—C4—C6	115.62 (19)	C11—C12—F1	118.4 (3)
C7—C6—C4	121.8 (2)	C12—C13—C14	119.0 (3)
C7—C6—S1	128.96 (18)	C12—C13—H13	120.5
C4—C6—S1	109.24 (16)	C14—C13—H13	120.5
C6—C7—C1	118.8 (2)	C1—O1—C17	118.1 (2)
C6—C7—H7	120.6	O1—C17—H17A	109.5
C1—C7—H7	120.6	O1—C17—H17B	109.5
O1—C1—C2	124.3 (2)	H17A—C17—H17B	109.5
O1—C1—C7	115.5 (2)	O1—C17—H17C	109.5
C2—C1—C7	120.2 (2)	H17A—C17—H17C	109.5
C4—C3—C2	120.3 (2)	H17B—C17—H17C	109.5
C4—C3—H3	119.8	C16—O4—P1	123.11 (18)
C2—C3—H3	119.8	C15—O3—P1	121.1 (2)
C1—C2—C3	120.5 (2)	O4—C16—H16A	109.5
C1—C2—H2	119.8	O4—C16—H16B	109.5
C3—C2—H2	119.8	H16A—C16—H16B	109.5
C5—N1—C8	121.96 (19)	O4—C16—H16C	109.5
C5—N1—H1	119.0	H16A—C16—H16C	109.5
C8—N1—H1	119.0	H16B—C16—H16C	109.5
N1—C8—C9	115.05 (18)	O3—C15—H15A	109.5
N1—C8—P1	107.14 (15)	O3—C15—H15B	109.5
C9—C8—P1	110.28 (15)	H15A—C15—H15B	109.5
N1—C8—H8	108.0	O3—C15—H15C	109.5
C9—C8—H8	108.0	H15A—C15—H15C	109.5
P1—C8—H8	108.0	H15B—C15—H15C	109.5
C14—C9—C10	118.3 (2)		

### Hydrogen-bond geometry (Å, °)

**Table:**

*D*—H···*A*	*D*—H	H···*A*	*D*···*A*	*D*—H···*A*
N1—H1···O2^i^	0.86	1.99	2.793 (3)	156
C8—H8···N2	0.98	2.44	2.868 (4)	106
C14—H14···N1	0.93	2.62	2.919 (5)	100
C16—H16C···F1^ii^	0.96	2.51	3.2496	133
C15—H15B···Cg1^iii^	0.96	2.99	3.608 (4)	123
C16—H16B···Cg3^iv^	0.96	2.96	3.549 (4)	121
C17—H17C···Cg1^v^	0.96	2.88	3.625 (3)	136

Symmetry codes: (i) −*x*+1/2, −*y*+1/2, −*z*;  (ii) −*x*+1, *y*, −*z*+1/2; (iii) −*x*, *y*, −*z*−1/2; (iv) −*x*+1/2, *y*+1/2, −*z*−1/2; (v) −*x*, *y*, −*z*+1/2.

## References

[bb1] Alvarez, R. G., Kennedy, A. R., Khalaf, A. I., Suckling, C. J. & Waigh, R. D. (2005). *Acta Cryst.* E**61**, o569–o570.

[bb2] Bruker (2004). *APEX2* Bruker AXS Inc., Madison, Wisconsin, USA.

[bb3] Chen, R. Y. & Li, Y. G. (1987). *Chemistry of Organic Phosphorus*, p.38. Beijing: High Education Press.

[bb4] De Lombaert, S., Blanchard, L., Tan, J., Sakane, Y., Berry, C. & Ghai, R. D. (1995). *Bioorg. Med. Chem. Lett.* **5**, 145–150.

[bb5] Du, S. C., Faiger, H., Belakhov, V. & Timor Bassov, T. (1999). *Bioorg. Med. Chem.* **7**, 2671–2682. 10.1016/s0968-0896(99)00233-3 10658571

[bb6] Fang, H., Fang, M.-J., Xu, Y., Yu, W.-C. & Zhao, Y.-F. (2009). *Acta Cryst.* E**65**, o642. 10.1107/S1600536809006382 PMC2968692 21582291

[bb7] Jin, L. H., Song, B. A., Zhang, G. P., Xu, R. Q., Zhang, S. M., Gao, X. W., Hu, D. Y. & Yang, S. (2006). *Bioorg. Med. Chem. Lett.* **16**, 1537–1543. 10.1016/j.bmcl.2005.12.041 16406612

[bb8] Kafarski, P. & Lejczak, B. (2001). *Curr. Med. Chem. Anti-Cancer Agents*, **1**, 301–312. 10.2174/1568011013354543 12678760

[bb9] Li, B., Zhang, S., Wang, Y. & Luo, S. (2008). *Acta Cryst.* E**64**, o1549. 10.1107/S1600536808021089 PMC2962173 21203253

[bb10] Sheldrick, G. M. (1996). *SADABS* University of Göttingen, Germany.

[bb11] Sheldrick, G. M. (2008). *Acta Cryst.* A**64**, 112–122. 10.1107/S0108767307043930 18156677

[bb12] Song, B. A., Chen, C. J., Yang, S., Jin, L. H., Xue, W., Zhang, S. M., Zou, Z. H., Hu, D. Y. & Liu, G. (2005). *Acta Chim. Sin* **63**, 1720–1726.

[bb13] Yang, S., Song, B. A., Hong, Y. P., Jin, L. H. & Hu, D. Y. (2005). J. Chem. Crystallogr. **11**, 891–895.

